# Functional and structural characterization of HspB1/Hsp27 from Chinese hamster ovary cells

**DOI:** 10.1002/2211-5463.12726

**Published:** 2019-09-04

**Authors:** Eiryo Sha, Manami Nakamura, Kazuya Ankai, Yohei Y. Yamamoto, Toshihiko Oka, Masafumi Yohda

**Affiliations:** ^1^ Department of Biotechnology and Life Science Tokyo University of Agriculture and Technology Japan; ^2^ Department of Physics Faculty of Science Shizuoka University Japan

**Keywords:** chaperone, CHO cell, folding, small heat shock protein, small‐angle X‐ray scattering, stress response

## Abstract

Small heat shock proteins (sHsps) endow cells with stress tolerance. Of the various sHsps in mammals, HspB1, also known as Hsp27, is the most ubiquitous. To examine the structure and function of HspB1, we expressed, purified, and characterized HspB1 from Chinese hamster (*Cricetulus griseus*) ovary cells (CgHspB1). CgHspB1 forms a large oligomeric structure. We observed a monodisperse 16‐mer with an elongated sphere, but this is affected by changes in various conditions, including temperature. Under dilute conditions, CgHspB1 dissociates into small oligomers at elevated temperatures. The dissociated conformers interacted with the gel filtration column through hydrophobic interactions. In contrast, dissociation of the oligomer was not observed by small‐angle X‐ray scattering at 55 °C. The result partially coincides with the results of size exclusion chromatography, showing that dissociation did not occur at high protein concentrations. However, a significant structural change in the oligomeric conformations appears to occur between room and higher temperatures. Reflecting their status as homeotherms, mammalian sHsps are regulated by phosphorylation. A phosphorylation mimic mutant of CgHspB1 with the replacement of Ser15 to Asp exhibited relatively lower oligomer stability and greater protective ability against thermal aggregation than the wild‐type protein. The result clearly shows a correlation between oligomer dissociation and chaperone activity.

**Abbreviations**
sHsps: small heat shock proteins
CHO: Chinese hamster ovary
CgHspB1: HspB1 from CHO cells
CgHspB1WT: wild‐type CgHspB1
CgHspB1S15D: CgHspB1 with S15D mutation
IPMDH: isopropyl malate dehydrogenase from *Thermus thermophilus* HB8
CS: porcine heart citrate synthase
SEC‐MALS: size exclusion chromatography–multiangle light scattering
SAXS: small‐angle X‐ray scattering

Small heat shock proteins (sHsps) endow cells with stress tolerance. sHsps bind to partially folded or denatured proteins, thereby preventing irreversible aggregation or promoting correct substrate folding 1, 2. Overall amino acid sequence homology between sHsps is considerably lower compared to other chaperones. Their common feature is the α‐crystallin domain which is named after the α‐crystallin in vertebrate lenses 3. The N‐terminal region is highly variable, and the C‐terminal extension is partially conserved with the consensus IXI motif 4. Most sHsps take large oligomeric structures composed of 12–36 subunits 5, 6, 7. The sHsp from *Methanocaldococcus jannaschii* (MjHsp16.5) forms a spherical 24‐mer oligomer with a diameter of 12 nm (PDB‐ID: 1SHS) 7. The sHsp of *Sulfolobus tokodaii* strain 7 (StHsp14.0) forms a similar oligomer composed of 24 subunits (PDB‐ID: 3VQK) 8. On the contrary, the sHsp from wheat (wHsp16.9) forms a double‐ring‐shaped oligomer consisting of 12 subunits (PDB‐ID: 1GME) 6. We have determined the crystal structure of a sHsp from the fission yeast, *Schizosaccharomyces pombe*, SpHsp16.0. SpHsp16.0 forms a hexadecameric oligomer structure in which eight dimers of SpHsp16.0 form an elongated sphere with 422 symmetry (PDB‐ID: 3W1Z) 9.

There exist 10 genes encoding sHsps in mammalian genomes 10. They differ slightly in monomeric molecular weight, stress inducibility, oligomeric structure, chaperone activity, and tissue distribution 11, 12, 13. HspB1/Hsp27 is almost ubiquitously expressed in all human tissues 11, 13 and is involved in the regulation of many vital functions. HspB1 seems to be responsible for regulation and stabilization of the cytoskeleton 14, 15, possesses anti‐apoptotic activity 16, 17, and protects the cell against oxidative stress 18, 19. Mammalian sHsps, which reflect the homeothermic status of mammals, are regulated by phosphorylation. Extracellular stresses induce phosphorylation two or three serine residues.

The molecular architecture of HspB1/Hsp27 is controversial. Analytical ultracentrifugation analysis showed that the mean molecular mass is 730 kDa 20. On the contrary, HspB1/Hsp27 in the nonphosphorylated state was reported to form 24‐mers by gel‐filtration chromatography studies 21. Lelj‐Garolla *et al*. 22 showed that HspB1/Hsp27 exists in the equilibrium state of monomers/dimers, tetramers, 12‐mers, and 16‐mers based on sedimentation velocity analysis. The same group has shown that oligomerization of HspB1/Hsp27 increases with the temperature elevation from 10 to 40 °C. The largest oligomers at 10 °C were 8–12‐mers, whereas oligomers as large as 22–30‐mers were observed at 40 °C 23. This observation contradicts the general knowledge that the large oligomeric structures of sHsps disassemble to smaller oligomers at the high temperature 24, 25. The analysis by size exclusion chromatography showed that the wild‐type HspB1/Hsp27 eluted as a broad peak with an average molecular mass of approximately 590 kDa 26. The molecular mass decreased by introducing phosphorylation mimic mutations. Chaperone activity is also increased by mutations. Therefore, it is reasonable to think that the dissociation of oligomers is correlated with molecular chaperone activity.

The crystal structure of the human HspB1 α‐crystallin domain has been reported 27. Unexpectedly, the HspB1 fragment does not form the typical β7/β7 dimers but rather hexamers by an asymmetric contact between the β4 and the β7 strands from the adjacent α‐crystallin domain.

In this study, we expressed and characterized HspB1/Hsp27 from Chinese hamster ovary (CHO) cells. According to the scientific name of the Chinese hamster, *Cricetulus griseus*, it is referred to as HspB1 from CHO cells (CgHspB1) hereafter. CHO cells are mostly used for industrial production of therapeutic proteins. Proteostasis in CHO cells should be important for the production of therapeutic proteins. However, there have been only a few reports on chaperones, including HspB1/Hsp27 in CHO cells. One of the advantages of CHO cells compared with other mammalian cells is its robustness. HspB1/Hsp27 is also known to play a role in the inhibition of apoptosis and actin cytoskeletal remodeling. Thus, it may take an essential role in the robustness of CHO cells.

We have firstly performed structural and functional characterization of HspB1/Hsp27 from CHO cell. The results will give the insights not only to the functional mechanism of HspB1/Hsp27 but also to the proteostasis and robustness of CHO cell.

## Materials and methods

### Cloning, expression, and purification

The full‐length gene for CgHspB1 was amplified from total cDNA of CHO cells using the primers 5′‐GGA TAT CCA TAT GAC CGA GCG CCG CG‐3′ and 5′‐GAA TTC CTA CTT GGC TCC AGA CTG TTC CGA CTT C‐3′. The amplified DNA fragment was digested with Nde I and EcoR I and inserted into the Nde I/EcoR I site of pET23b. Then, the constructed plasmid, pET23b‐wild‐type CgHspB1 (CgHspB1WT), was used for the production of CgHspB1WT in *Escherichia coli* BL21 Star (DE3). The plasmid for the production of the phosphorylated mimic CgHspB1, CgHspB1 with S15D mutation (CgHspB1S15D), was made through site‐directed mutagenesis with the primers 5′‐GCT GCT GCG GAG CCC CGA CTG GGA ACC ATT CCG GG‐3′ and 5′‐CCC GGA ATG GTT CCC AGT CGG GGC TCC GCA GCA GC‐3′ using pET23b‐ CgHspB1WT as a template 28.

*Escherichia coli* BL21 (DE3) cells transformed with pET23b‐CgHspB1WT or pET23b‐CgHspB1S15D were grown at 37 °C in Luria–Bertani medium containing 100 µg·mL^−1^ ampicillin for 24 h. The cells were harvested by centrifugation at 5000 ***g*** for 10 min at 4 °C.

The harvested cells were suspended in buffer A (50 mm Tris/HCl, pH 8.0) and disrupted by sonication, and the suspension of disrupted cells was centrifuged at 24 000 ***g*** for 30 min at 4 °C. The supernatant was applied to a TOYOPEARL DEAE‐650 anion exchange column (Tosoh, Tokyo, Japan) equilibrated with buffer A. Proteins were eluted with a linear gradient of 0–400 mm NaCl in buffer A. Fractions containing CgHspB1 were pooled and dialyzed with buffer A overnight. The dialyzed protein solution was applied to a RESOURCE Q column (GE Healthcare Bio‐Sciences, Buckinghamshire, UK) equilibrated with buffer A. Proteins were eluted with a linear gradient of 0–500 mm NaCl in buffer A. Fractions containing CgHspB1 were pooled, concentrated by ultrafiltration (Amicon Ultra, Merck Millipore, Billerica, CA, USA), and then applied to a HiLoad 26/60 Superdex 200 pg size exclusion column (GE Healthcare Bio‐Sciences) equilibrated with buffer B (50 mm Tris/HCl pH 7.5, 0.1 mm EDTA, 150 mm NaCl).

Isopropyl malate dehydrogenase from *Thermus thermophilus* HB8 (IPMDH) was expressed in *E. coli* and purified as described previously 29.

### Protein aggregation measurements

The thermal aggregation of porcine heart citrate synthase (CS) was monitored by measuring light scattering at 500 nm with a spectrofluorometer (FP‐6500; JASCO, Tokyo, Japan) at 45 °C as described previously 30. Native CS (50 nm, monomer) was incubated in TKM buffer (50 mm Tris/HCl, pH 7.5, 100 mm KCl, and 25 mm MgCl_2_) with or without CgHspB1WT or CgHspB1S15D. The assay buffer was preincubated at 45 °C and continuously stirred throughout the measurement.

### Size exclusion chromatography

Size exclusion chromatography was performed with a gel‐filtration column (SB‐804HQ; Showa Denko, Tokyo, Japan) using an HPLC system, PU‐1580i, connected to a MD1515 multiwavelength detector (JASCO) as described previously 31. CgHspB1WT or CgHspB1S15D was diluted to the specified concentrations (as monomer) in buffer B. A 100‐µL aliquot of diluted CgHspB1WT or CgHspB1S15D was heated at the specified temperature for 30 min and then loaded onto a column heated at the same temperature and eluted with buffer B with or without 20% ethylene glycol at a flow rate of 1.0 mL·min^−1^. The proteins are monitored by the absorbance at 215 nm. To examine the reversibility of the dissociation, CgHspB1WT or CgHspB1S15D preheated at 45 °C for 30 min was analyzed by gel filtration at room temperature after cooling at 25 °C for 30 min.

### Size exclusion chromatography–multiangle light scattering

The purified CgHspB1 was analyzed by size exclusion chromatography–multiangle light scattering (SEC‐MALS) on a TSKgel G3000XL column (Tosoh) connected to a multiangle light‐scattering detector (MINI DAWN; Wyatt Technology, Santa Barbara, CA, USA) and a differential refractive index detector (Shodex RI‐101; Showa Denko) with an HPLC system, PU‐980i (JASCO), as described previously 31. A 100‐µL aliquot of sample was injected into the column and eluted with buffer B at 1.0 mL·min^−1^. The molecular weight and protein concentration were determined according to the instructional manual (Wyatt Technology).

### SAXS measurements

Small‐angle X‐ray scattering (SAXS) was performed on a laboratory system (NANO‐Viewer system; Rigaku, Tokyo, Japan). The two‐dimensional scattering data were measured using a two‐dimensional detector (PILATUS 100K; Dectris, Baden, Switzerland), and the data were circularly averaged to one‐dimensional data. The sample detector distance was set to 791 mm, which was calibrated with silver behenate. The scattering intensity, *I*(*Q*), was measured for scattering vectors (*Q* = 4π sinθ/λ) ranging from 0.012 to 0.2 Å^−1^. The temperature was maintained at 25 °C or 55 °C. The innermost part of *I*(*Q*) was fitted under the Guinier approximation 32 to the equation *I*(*Q*) = *I*(0)exp[−*R*_g_^2^ *Q* ^2^/3], where *I*(0) and *R*_g_ are the forward scattering intensity (*Q* = 0) and the radius of gyration, respectively. A series of diluted samples were measured to extrapolate *C*/*I*(0) and *R*_g_^2^ to zero protein concentration. The sample concentration ranged from 0.56 to 9.93 mg·mL^−1^. The low‐resolution model was constructed from the SAXS data at 25 °C by DAMMIF 33 without symmetrical constraints. Ten independent models were averaged by DAMAVER 34. Figures of the low‐resolution model were prepared using the pymol program 35.

## Results

**Figure 1:**
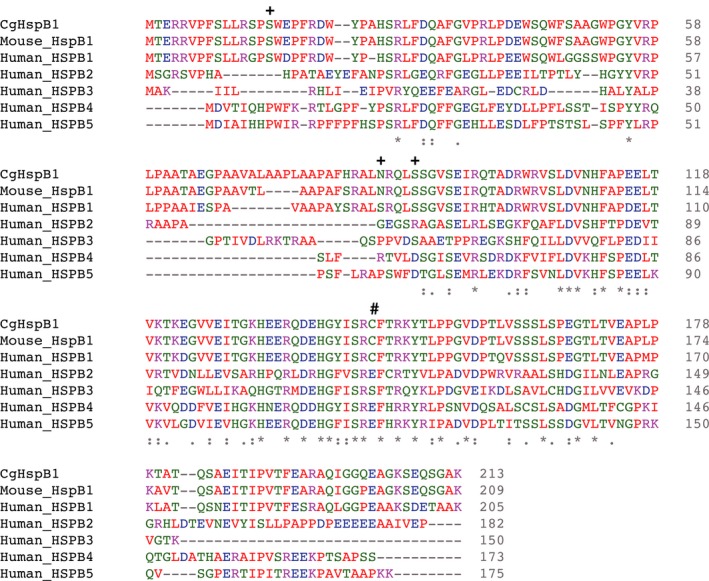
Amino acid sequence alignment of Chinese hamster HspB1 with various sHsps. Amino acid sequence alignment of human HspB1 (HUMAN_HSPB1, P04792), Chinese hamster HspB1 (CgHSPB1), mouse HspB1 (MOUSE_HSPB1, P14602), human HspB2 (HUMAN_HSPB2, Q16082), human HspB3 (HUMAN_HSPB3, Q12988), human HspB4 (HUMAN_HSPB4, P02489), and human HspB5 (HUMAN_HSPB5, P02511) is shown. The three phosphorylated Ser residues in Human HspB1 are marked by ‘+’. The Cys residue that forms inter‐subunit disulfide bond is marked by ‘#’.

We amplified full‐length cDNA for HspB1/Hsp27 from total cDNA of CHO cells using PCR. The amino acid sequence of CHO HspB1/Hsp27 (CgHspB1) was almost identical to those of other mammals (Fig. 1). Among three putative phosphorylation sites, Ser 15 and Ser 82 of human HspB1 are conserved in CgHspB1. It is known that murine and human HspB1 has only one cysteine residue, and the dimeric unit is connected by a disulfide bond 36, 37. Although the disulfide bond is not indispensable for dimer formation, it is thought to be related to the regulation of HspB1 by oxidative stress. The cysteine residue is also conserved in CgHspB1.

**Figure 2:**
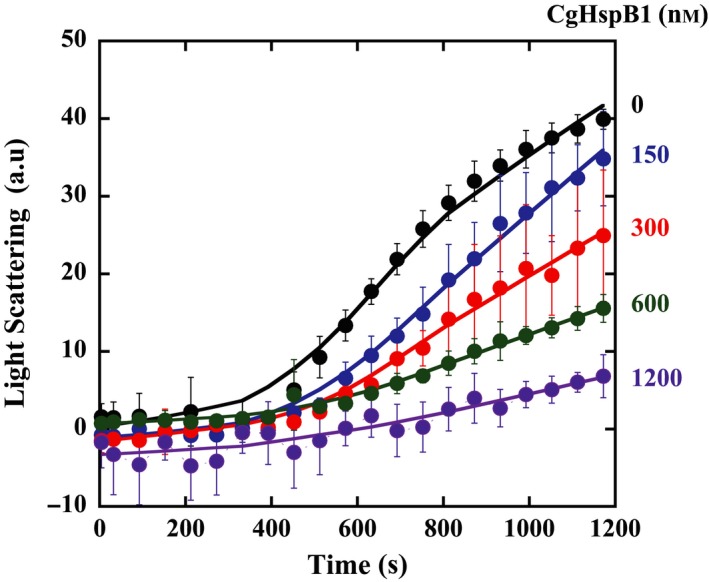
Effect of CgHspB1WT on thermal aggregation of CS. The thermal aggregation of CS from porcine heart was monitored by measuring light scattering at 500 nm with a spectrofluorometer at 45 °C. CS (50 nm, monomer) was incubated in the assay buffer with or without CgHspB1WT (150, 300, 600, and 1200 nm as monomers). The average values with the error bars of standard deviations from triplicate assays are plotted.

Wild‐type CgHspB1 was expressed in *E. coli* and purified to homogeneity. First, we examined the chaperone activity of CgHspB1WT. CgHspB1WT protected CS from thermal aggregation at 45 °C (Fig. 2). Near‐complete suppression of aggregation was attained by the addition of 24 excess molar CgHspB1WT.

**Figure 3:**
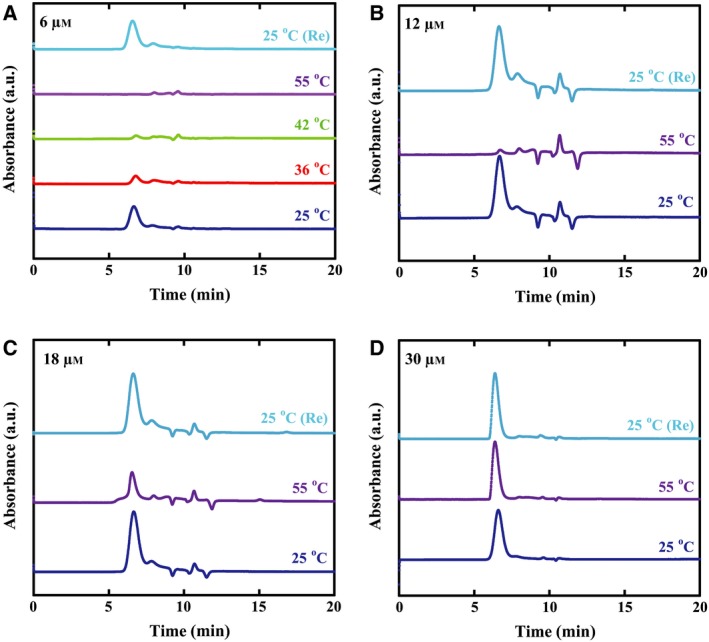
Oligomer dissociation of CgHspB1 at elevated temperatures. CgHspB1WT (A. 6 µm; B. 12 µm; C. 18 µm; D. 30 µm as monomer) was incubated at the specified temperature for 30 min and then analyzed using size exclusion chromatography at the same temperature. CgHspB1WT heated to 55 °C was analyzed by gel filtration at room temperature after cooling at 25 °C for 30 min [25 °C(Re)].

Then, we examined the temperature‐ and concentration‐dependent conformational change of CgHspB1WT using size exclusion chromatography on an HPLC system (Fig. 3). CgHspB1WT exists as a large oligomer similar to other sHsps. From the retention time, the molecular weight is estimated to be larger than 100 kDa.

**Figure 4:**
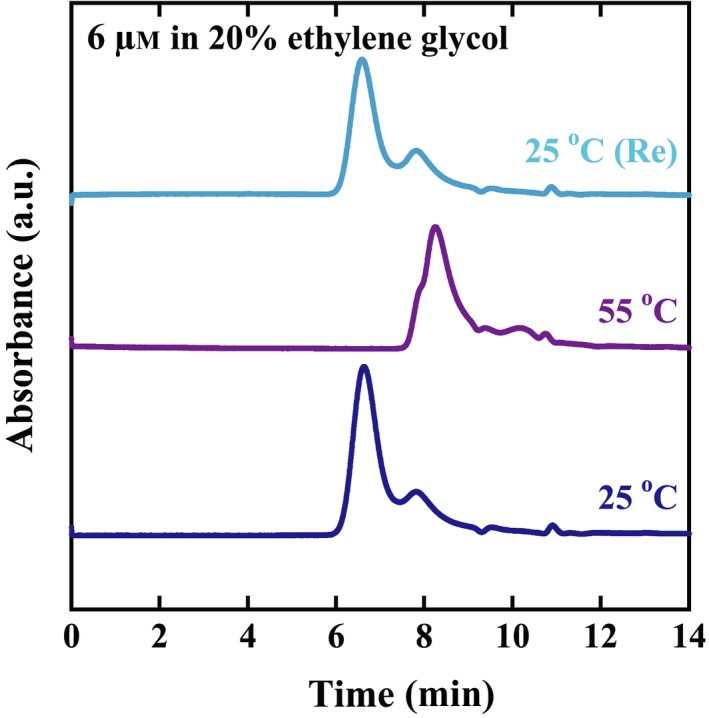
Oligomer dissociation of CgHspB1 analyzed by gel filtration in the presence of 20% ethylene glycol. CgHspB1WT (6 µm) was analyzed by gel filtration using buffer containing 20% ethylene glycol.

The CgHspB1WT oligomer decreased at the elevated temperature, and the oligomer reappeared when the temperature was shifted to the room temperature (Fig. 3A). Curiously, peaks for the dissociated small oligomers or monomers were not observed at the elevated temperatures (Fig. 3B–D). When the concentration was 6 µm, the oligomer peak completely disappeared, and only trace broad peaks for smaller oligomers appeared. The oligomer dissociation was also dependent on concentration. Almost no change was observed when the concentration was 30 µm (Fig. 3D). To examine the conformation of CgHspB1WT at the elevated temperature, size exclusion chromatography was performed with a buffer containing 20% ethylene glycol (Fig. 4) 31. As ethylene glycol reduces hydrophobic interactions, nonspecific interactions between CgHspB1WT and the column resin should be reduced. Under these conditions, we observed the peak for CgHspB1WT at a position for small oligomers. Since the retention time of the peak corresponds to that for the polymers with the molecular weights of several ten kDa, they seem to be dimers. The results suggest that CgHspB1WT dissociates into dimers at elevated temperature and the hydrophobic surface is exposed.

**Figure 5:**
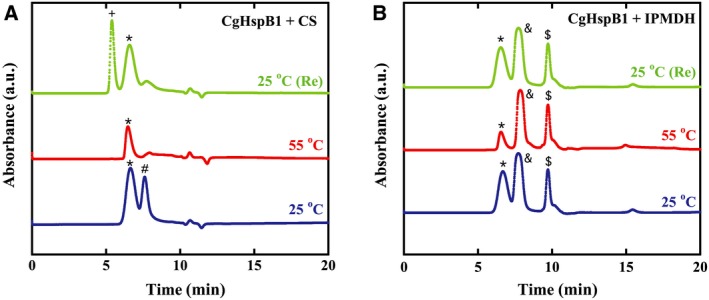
Interaction of CgHspB1 with client proteins. CgHspB1WT (30 µm) was incubated with CS (A) or IPMDH (B) at 25 and 55 °C for 30 min and then analyzed by size exclusion chromatography at the same temperature. The mixture incubated at 55 °C was cooled to 25 °C and analyzed at 25 °C (25 °C Re).(A) CgHspB1 + CS. +, complex of CgHspB1 and CS; *, CgHspB1 oligomer; #, CS. (B) CgHspB1 + IPMDH. * CgHspB1 oligomer; &, IPMDH dimer; $, IPMDH monomer.

The mixtures of CgHspB1 and client proteins were analyzed with size exclusion chromatography (Fig. 5). CgHspB1WT (30 µm) and CS appeared as separate peaks at room temperature. At 55 °C, the peak for CgHspB1WT remained, but the peak for CS disappeared. The oligomer peaks stayed in the same position. It is reasonable to think that not the original large oligomer but the dissociated small oligomers interact with the denatured CS. Then, the heated mixture was cooled and analyzed at room temperature. The large complex of CgHspB1 and CS appeared. The same experiment was performed using a thermostable protein, IPMDH (Fig. 5). Since IPMDH does not denature at 55 °C, the presence of IPMDH did not affect CgHspB1.

**Figure 6:**
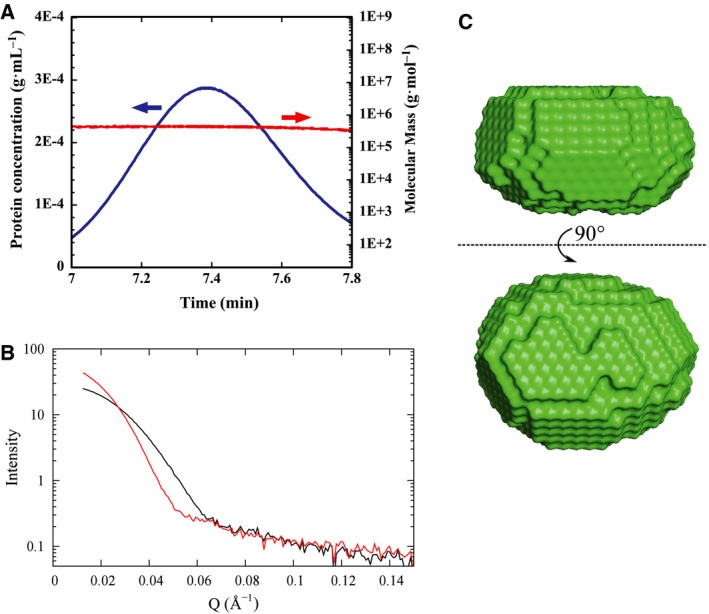
Oligomeric structure of CgHspB1 analyzed by SAXS. (A) Molecular mass determination of CgHspB1 by SEC‐MALS. The purified CgHspB1 was analyzed by SEC‐MALS on a TSKgel G3000XL column connected to a multiangle light‐scattering detector (red) and a differential refractive index detector (blue) on an HPLC system, PU‐980i. (B) SAXS profile of CgHspB1. SAXS profiles of CgHspB1 25 °C (black) or 55 °C (red) are shown. (C) Structure of CgHspB1 oligomer calculated from SAXS data. The SAXS envelope of CgHspB1 was calculated from the SAXS profile at 25 °C.

The molecular mass of CgHspB1WT at room temperature was determined to be 384 kDa using SEC‐MALS (Fig. 6A). As the deduced molecular mass of a subunit is 23.4 kDa, the oligomer is calculated to be a 16‐mer. The oligomeric state of CgHspB1WT was also investigated with SAXS (Fig. 6B). *R*_g_ at 25 °C was estimated to be 60.9 Å, and the molecular mass calculated from the *I*(0) value was 361 kDa, which was almost the same as that calculated from the results of SEC‐MALS. The low‐resolution model of the oligomer of CgHspB1WT in solution was constructed from the SAXS data at 25 °C (Fig. 6C). The SAXS model was an oval sphere, which is similar to the crystal structure of SpHsp16.0 9. Curiously, dissociation of the oligomer was not observed by SAXS. At 55 °C, the *R*_g_ value increased to 75.3 Å (Fig. 6B). The results partly coincide with the size exclusion chromatography results in which dissociation was not observed at high protein concentrations, because the SAXS experiment is performed at a high protein concentration.

**Figure 7:**
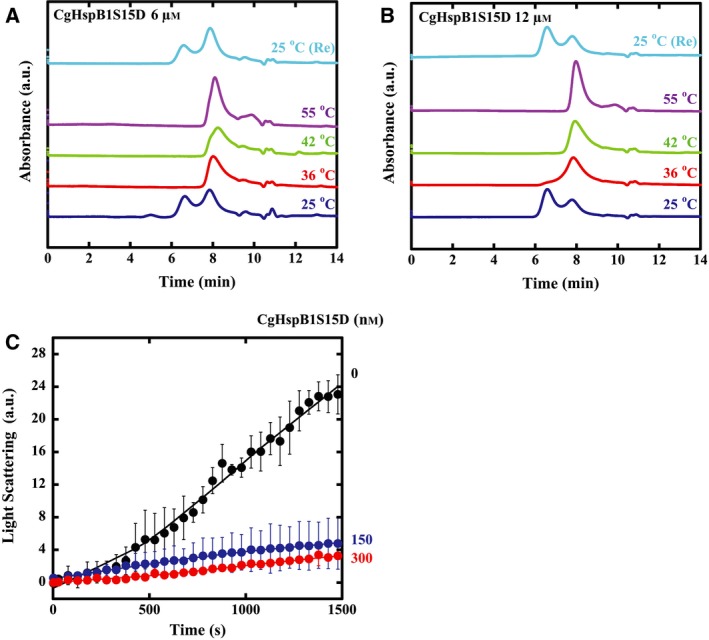
Characterization of the phosphorylation mimic mutant of CgHspB1 and CgHspBS15D. (A, B) Oligomer dissociation of CgHspBS15D at elevated temperatures. CgHspBS15D (A. 6 µm; B. 12 µm) was incubated at the specified temperature for 30 min and then analyzed with size exclusion chromatography using a buffer containing 20% ethylene glycol at the same temperature. CgHspBS15D heated to 45 °C was analyzed by gel filtration at room temperature after cooling at 25 °C for 30 min [25 °C (Re)]. (C) Effect of CgHspBS15D on the thermal aggregation of CS. The thermal aggregation of CS from the porcine heart was monitored by measuring light scattering at 500 nm with a spectrofluorometer at 45 °C. Native CS (50 nm, monomer) was incubated in assay buffer with or without CgHspBS15D (150 and 300 nm as monomers). The average values with the error bars of standard deviations from duplicate assays are plotted.

Among the putative phosphorylation sites of HspB1, two are conserved in CgHspB1 (Fig. 1). Since Ser15 is conserved only in HspB1, we focused Ser15 and examined the effect of phosphorylation by analyzing S15D mutant. Compared with the wild‐type, the CgHspB1S15D oligomer is unstable (Fig. 7A). Even at the room temperature, CgHspB1S15D was partially dissociated into small oligomers, likely dimers (Fig. 7A,B). CgHspB1S15D showed a significantly high capacity to protect CS from thermal aggregation (Fig. 7C). An equimolar amount of CgHspB1S15D was sufficient to suppress the increase in light scattering induced by aggregation of CS.

## Discussion

We have performed functional and structural characterization of HspB1 from Chinese ovary cell (CgGspB1). CgHspB1 could suppress the thermal aggregation of CS. CgHspB1 exists as a large oligomer and exhibits temperature‐dependent dissociation. The dissociation also depends on the concentrations. Curiously, almost no change was observed at high concentrations. SAXS experiments also showed that CgHspB1 remained as the large oligomer at high temperature.

The molecular architecture of HspB1 is controversial. Analytical ultracentrifugation showed that the mean molecular mass is 730 kDa 20, and gel‐filtration chromatography studies indicated that HspB1 forms 24‐mers in the nonphosphorylated state 21. Our result coincides with the observation by Lelj‐Garolla *et al*. 22 that HspB1 exists as an equilibrium mixture of monomers/dimers, tetramers, 12‐mers, and 16‐mers based on sedimentation velocity analysis. Analytical ultracentrifugation experiments with various HspB1 concentrations clearly demonstrate that the oligomeric size increases from 10 to 40 °C. These larger oligomers are in equilibrium with smaller species, and their association is reversible. Therefore, they are not nonspecific aggregates 22. Although we have shown that CgHspB1 exits as 16‐mer structure, it seems to be variable as there is a difference in the oligomeric structures between at the room temperature and the elevated temperature.

Rogalla *et al*. 21 demonstrated that both phosphorylated HspB1 and the phosphorylation mimic mutant showed significantly decreased chaperone activity *in vitro*. They concluded that large oligomers of sHsps are necessary for chaperone action. However, other studies have clearly shown that phosphorylation mimic mutations destabilize HspB1/Hsp27 oligomers and enhance chaperone activity 26.

We have shown that a single phosphorylation mimic at S15 significantly increased chaperone activity and decreased oligomer stability. Even at the room temperature, CgHspB1S15D partially dissociated into small oligomers, likely dimers (Fig. 7A,B).

The hydrophobic character of the dissociated dimers was clearly shown by the interaction with the gel‐filtration column. The correlation between chaperone activity and oligomer dissociation is clearly shown by the comparison between CgHspB1WT and CgHspB1S15D. However, the idea is contradicting with the fact that dissociation of CgHspB1WT was not observed at the relatively high concentration. The discrepancy can be explained as follows. CgHspB1 is in the dynamic equilibrium between large oligomers and small oligomers. In the large oligomeric conformation, the hydrophobic surface remains inside. The hydrophobic surfaces are exposed by dissociation of the oligomers to interact with unfolded polypeptides. At a high concentration, most of the CgHspB1 remains in the large oligomeric conformation. However, the large oligomers are not static. They exchange dimer units which interact with unfolded proteins. Thus, the hydrophobic surface of the large oligomers is occasionally exposed by releasing a dimer unit, which may induce the formation of various oligomeric structures.

## Conflicts of interest

The authors declare no conflict of interest.

## Author contributions

MY conceived and designed the experiments. ES, MN, KA, YYY, and TO performed the experiments. YYY, TO, and MY analyzed the data. ES, MN, KA, YYY, TO, and MY contributed reagents/materials/analysis tools. MY wrote the manuscript.

## References

[1] van den IJssel PR , Overkamp P , Knauf U and Gaestel M and de Jong WW (1994) Alpha A‐crystallin confers cellular thermoresistance. FEBS Lett 355, 54–56. 7957962 10.1016/0014-5793(94)01175-3

[2] Jakob U , Gaestel M , Engel K and Buchner J (1993) Small heat shock proteins are molecular chaperones. J Biol Chem 268, 1517–1520. 8093612

[3] Caspers GJ , Leunissen JA and de Jong WW (1995) The expanding small heat‐shock protein family, and structure predictions of the conserved "alpha‐crystallin domain". J Mol Evol 40, 238–248. 7723051 10.1007/BF00163229

[4] Pasta SY , Raman B , Ramakrishna T and Rao ChM (2002) Role of the C‐terminal extensions of alpha‐crystallins. Swapping the C‐terminal extension of alpha‐crystallin to alphaB‐crystallin results in enhanced chaperone activity. J Biol Chem 277, 45821–45828. 12235146 10.1074/jbc.M206499200

[5] Bepperling A , Alte F , Kriehuber T , Braun N , Weinkauf S , Groll M , Haslbeck M and Buchner J (2012) Alternative bacterial two‐component small heat shock protein systems. Proc Natl Acad Sci USA 109, 20407–20412. 23184973 10.1073/pnas.1209565109 PMC3528540

[6] van Montfort RL , Basha E , Friedrich KL , Slingsby C and Vierling E (2001) Crystal structure and assembly of a eukaryotic small heat shock protein. Nat Struct Biol 8, 1025–1030. 11702068 10.1038/nsb722

[7] Kim KK , Kim R and Kim SH (1998) Crystal structure of a small heat‐shock protein. Nature 394, 595–599. 9707123 10.1038/29106

[8] Hanazono Y , Takeda K , Yohda M and Miki K (2012) Structural studies on the oligomeric transition of a small heat shock protein, StHsp14.0. J Mol Biol 422, 100–108. 22613762 10.1016/j.jmb.2012.05.017

[9] Hanazono Y , Takeda K , Oka T , Abe T , Tomonari T , Akiyama N , Aikawa Y , Yohda M and Miki K (2013) Nonequivalence observed for the 16‐meric structure of a small heat shock protein, SpHsp16.0, from *Schizosaccharomyces pombe* . Structure 21, 220–228. 23273429 10.1016/j.str.2012.11.015

[10] Haslbeck M , Franzmann T , Weinfurtner D and Buchner J (2005) Some like it hot: the structure and function of small heat‐shock proteins. Nat Struct Mol Biol 12, 842–846. 16205709 10.1038/nsmb993

[11] Taylor RP and Benjamin IJ (2005) Small heat shock proteins: a new classification scheme in mammals. J Mol Cell Cardiol 38, 433–444. 15733903 10.1016/j.yjmcc.2004.12.014

[12] Arrigo AP and Gibert B (2013) Protein interactomes of three stress inducible small heat shock proteins: HspB1, HspB5 and HspB8. Int J Hyperthermia 29, 409–422. 23697380 10.3109/02656736.2013.792956

[13] Mymrikov EV , Seit‐Nebi AS and Gusev NB (2011) Large potentials of small heat shock proteins. Physiol Rev 91, 1123–1159. 22013208 10.1152/physrev.00023.2010

[14] Mounier N and Arrigo AP (2002) Actin cytoskeleton and small heat shock proteins: how do they interact? Cell Stress Chaperones 7, 167–176. 12380684 10.1379/1466-1268(2002)007<0167:acashs>2.0.co;2 PMC514814

[15] Wettstein G , Bellaye PS , Micheau O and Bonniaud P (2012) Small heat shock proteins and the cytoskeleton: an essential interplay for cell integrity? Int J Biochem Cell Biol 44, 1680–1686. 22683760 10.1016/j.biocel.2012.05.024

[16] Acunzo J , Katsogiannou M and Rocchi P (2012) Small heat shock proteins HSP27 (HspB1), alphaB‐crystallin (HspB5) and HSP22 (HspB8) as regulators of cell death. Int J Biochem Cell Biol 44, 1622–1631. 22521623 10.1016/j.biocel.2012.04.002

[17] Garrido C , Paul C , Seigneuric R and Kampinga HH (2012) The small heat shock proteins family: the long forgotten chaperones. Int J Biochem Cell Biol 44, 1588–1592. 22449631 10.1016/j.biocel.2012.02.022

[18] Arrigo AP , Simon S , Gibert B , Kretz‐Remy C , Nivon M , Czekalla A , Guillet D , Moulin M , Diaz‐Latoud C and Vicart P (2007) Hsp27 (HspB1) and alphaB‐crystallin (HspB5) as therapeutic targets. FEBS Lett 581, 3665–3674. 17467701 10.1016/j.febslet.2007.04.033

[19] Christians ES , Ishiwata T and Benjamin IJ (2012) Small heat shock proteins in redox metabolism: implications for cardiovascular diseases. Int J Biochem Cell Biol 44, 1632–1645. 22710345 10.1016/j.biocel.2012.06.006 PMC3412898

[20] Behlke J , Lutsch G , Gaestel M and Bielka H (1991) Supramolecular structure of the recombinant murine small heat shock protein hsp25. FEBS Lett 288, 119–122. 1879544 10.1016/0014-5793(91)81016-2

[21] Rogalla T , Ehrnsperger M , Preville X , Kotlyarov A , Lutsch G , Ducasse C , Paul C , Wieske M , Arrigo AP , Buchner J *et al* (1999) Regulation of Hsp27 oligomerization, chaperone function, and protective activity against oxidative stress/tumor necrosis factor alpha by phosphorylation. J Biol Chem 274, 18947–18956. 10383393 10.1074/jbc.274.27.18947

[22] Lelj‐Garolla B and Mauk AG (2005) Self‐association of a small heat shock protein. J Mol Biol 345, 631–642. 15581903 10.1016/j.jmb.2004.10.056

[23] Lelj‐Garolla B and Mauk AG (2006) Self‐association and chaperone activity of Hsp27 are thermally activated. J Biol Chem 281, 8169–8174. 16436384 10.1074/jbc.M512553200

[24] Haslbeck M , Walke S , Stromer T , Ehrnsperger M , White HE , Chen S , Saibil HR and Buchner J (1999) Hsp26: a temperature‐regulated chaperone. EMBO J 18, 6744–6751. 10581247 10.1093/emboj/18.23.6744 PMC1171736

[25] Hirose M , Tohda H , Giga‐Hama Y , Tsushima R , Zako T , Iizuka R , Pack C , Kinjo M , Ishii N and Yohda M (2005) Interaction of a small heat shock protein of the fission yeast, *Schizosaccharomyces pombe*, with a denatured protein at elevated temperature. J Biol Chem 280, 32586–32593. 16055437 10.1074/jbc.M504121200

[26] Jovcevski B , Kelly MA , Rote AP , Berg T , Gastall HY , Benesch JL , Aquilina JA and Ecroyd H (2015) Phosphomimics destabilize Hsp27 oligomeric assemblies and enhance chaperone activity. Chem Biol 22, 186–195. 25699602 10.1016/j.chembiol.2015.01.001

[27] Baranova EV , Weeks SD , Beelen S , Bukach OV , Gusev NB and Strelkov SV (2011) Three‐dimensional structure of alpha‐crystallin domain dimers of human small heat shock proteins HSPB1 and HSPB6. J Mol Biol 411, 110–122. 21641913 10.1016/j.jmb.2011.05.024

[28] Fisher CL and Pei GK (1997) Modification of a PCR‐based site‐directed mutagenesis method. Biotechniques 23, 570 – 571, 574. 9343663 10.2144/97234bm01

[29] Okochi M , Matsuzaki H , Nomura T , Ishii N and Yohda M (2005) Molecular characterization of the group II chaperonin from the hyperthermophilic archaeum Pyrococcus horikoshii OT3. Extremophiles 9, 127–134. 15538645 10.1007/s00792-004-0427-y

[30] Abe T , Oka T , Nakagome A , Tsukada Y , Yasunaga T and Yohda M (2011) StHsp14.0, a small heat shock protein of *Sulfolobus tokodaii* strain 7, protects denatured proteins from aggregation in the partially dissociated conformation. J Biochem 150, 403–409. 21659385 10.1093/jb/mvr074

[31] Sugino C , Hirose M , Tohda H , Yoshinari Y , Abe T , Giga‐Hama Y , Iizuka R , Shimizu M , Kidokoro S , Ishii N *et al* (2009) Characterization of a sHsp of *Schizosaccharomyces pombe*, SpHsp15.8, and the implication of its functional mechanism by comparison with another sHsp, SpHsp16.0. Proteins 74, 6–17. 18543332 10.1002/prot.22132

[32] Guinier A and Fournet G (1955) Small Angle Scattering of X‐rays. John Wiley and Sons, New York, NY.

[33] Franke D and Svergun DI (2009) DAMMIF, a program for rapid ab‐initio shape determination in small‐angle scattering. J Appl Crystallogr 42, 342–346. 27630371 10.1107/S0021889809000338 PMC5023043

[34] Volkov VV and Svergun DI (2003) Uniqueness of Ab initio shape determination in small‐angle scattering. J Appl Crystallogr, 36, 860–864. 10.1107/S0021889809000338 PMC5023043 27630371

[35] DeLano WL (2002) The PyMOL Molecular Graphics System. DeLano Scientific LLC, San Carlos, CA.

[36] Chalova AS , Sudnitsyna MV , Semenyuk PI , Orlov VN and Gusev NB (2014) Effect of disulfide crosslinking on thermal transitions and chaperone‐like activity of human small heat shock protein HspB1. Cell Stress Chaperones 19, 963–972. 24898092 10.1007/s12192-014-0520-9 PMC4389837

[37] Zavialov A , Benndorf R , Ehrnsperger M , Zav'yalov V , Dudich I , Buchner J and Gaestel M (1998) The effect of the intersubunit disulfide bond on the structural and functional properties of the small heat shock protein Hsp25. Int J Biol Macromol 22, 163–173. 9650071 10.1016/s0141-8130(98)00014-2

